# Using Porcine Cadavers as an Alternative to Human Cadavers for Teaching Minimally Invasive Spinal Fusion: Proof of Concept and Anatomical Comparison

**DOI:** 10.7759/cureus.6158

**Published:** 2019-11-14

**Authors:** Hamid Abbasi, Ali Abbasi

**Affiliations:** 1 Neurosurgery, Inspired Spine Health, Minneapolis, USA; 2 Internal Medicine, Pritzker School of Medicine, Chicago, USA

**Keywords:** surgical training alternatives, spinal fusion, mis surgery, minimally invasive spinal surgery

## Abstract

Training surgeons to perform minimally invasive spinal (MIS) surgery is difficult because there are few realistic alternatives to human cadavers which are expensive and require special handling. In this study we report a protocol for performing an MIS training course on a fresh porcine cadaver. We find that the porcine lumbar spine closely resembles the human spine in terms of the vertebral and discal anatomy. Notable differences include a lower disc height and shallower diameter. We obtained fresh porcine cadavers weighing 40-70 kg from local farmers that had been gutted and bled. We position the cadaver prone on a backboard and set up the operating room with biplanar fluoroscopy. During approach and cage insertion, we found that the tactile feedback obtained is realistic and allows surgeons to familiarize themselves with the procedure. Porcine cadavers were also an excellent tool for practicing pedicle screw fixation due to the larger pedicles. Five training courses involving eight surgeons noted that except for anatomical differences the training course was equivalent to training on human cadavers and unanimously preferred training on porcine cadavers to synthetic foam models. We conclude that porcine cadavers are a useful model for training surgeons in MIS surgery. Routine use of porcine cadavers may increase the availability of MIS surgery training.

## Introduction

Minimally invasive spinal (MIS) surgery approaches have recently gained popularity because they are associated with significantly reduced tissue damage, complication rates and hospital stay. However, MIS surgeries can be difficult to master technically. In some procedures like MIS-TLIF (transforaminal lumbar interbody fusion), surgeons must perform the same procedure as in open operations through a smaller incision [1]. In other techniques like extreme lateral interbody fusion (XLIF) where surgeons rely solely on fluoroscopy and electrophysiology, surgeons must learn a completely new operation [2]. To learn these challenging techniques, surgeons must train extensively to master these procedures and continue training after the initial learning phase. These significant technical challenges associated with MIS operations may explain why MIS fusion has not gained widespread popularity and open surgeries remain the most frequently performed surgeries of the spine.

Unlike abdominal or thoracic surgery, spine surgery lacks a natural cavity within which endoscopic techniques can be utilized. This makes it extremely difficult to design simulators or artificial models for training surgeons on new MIS procedures. Simulators with the needed attributes do not yet exist to our best knowledge, which in turn slows down the adaptation of MIS techniques even though MIS may offer significant benefits to patients compared to open procedures. The ideal model beside a human cadaver is a biological model that can simulate human anatomy.

The standard method of training surgeons on new spinal operations remains a human cadaver model [3]. However, the supply of human cadavers is limited, expensive, and handling is complicated, limiting the availability of MIS training courses and reducing the hands-on experience of surgeons and trainees. Considering that surgeons must typically perform operations many times before becoming comfortable with them, cadaver labs are not a feasible option in most cases [2]. As an alternative to cadavers, some training centers use synthetic foam models. However, the consistency of foam differs substantially from human tissue making it an unrealistic alternative for learning procedures where tactile feedback is essential for success of the operation. These models typically lack anatomical soft tissue layers and appear drastically simplified on fluoroscopy, which does not allow the surgeon to become comfortable with the range of anatomical differences and pathologies that exists in human patients [4].

To overcome this challenge, we hypothesized that a fresh porcine cadaver may be a useful tool to train surgeons on MIS techniques. The common swine is one of the most commonly used species due to its easy availability and their similar size to humans. The porcine spine has also been used as a model for the human spine in biomechanical studies for testing new implants and devices [5-7]. The lumbar pedicles are known to be very similar in terms of size to the human equivalent, allowing surgeons to practice placing pedicle screws on porcine models [8,9]. The porcine spine is also similar to the human spine in terms of the shape of the endplates and spinal canal [10]. However, due to the different mode of weight bearing in pigs, the intervertebral discs height is significantly lower and the endplates are much smaller than in humans. Although cervical lordosis is similar, pigs have much lower thoracic kyphosis and lumbar lordosis than human spines [10].

In this study, we report on a protocol for performing MIS surgery training using a porcine cadaver, highlight the most important anatomical differences as they pertain to lumbar fusions, and present limited anecdotal feedback from participants of five training sessions. We performed these training sessions to teach surgeons a new technique for fusion of the lumbar spine known as trans-Kambin Oblique Lateral-Posterior Lumbar Interbody Fusion (OLLIF) [2]. In OLLIF, the disc space is approached through Kambin’s triangle and fusion is performed without direct visualization, guided only by bi-planar fluoroscopy, electrophysiology and tactile feedback. OLLIF is typically complemented with minimally invasive pedicle screw fixation, which we also performed on the porcine model. Cadaveric models have previously been described for practicing laminectomy, pedicle screw fixation, and lumbar puncture [11,12]. This study is the first to evaluate animal cadaveric models for simulating minimally invasive fusions of the lumbar spine.

## Materials and methods

**Figure 1:**
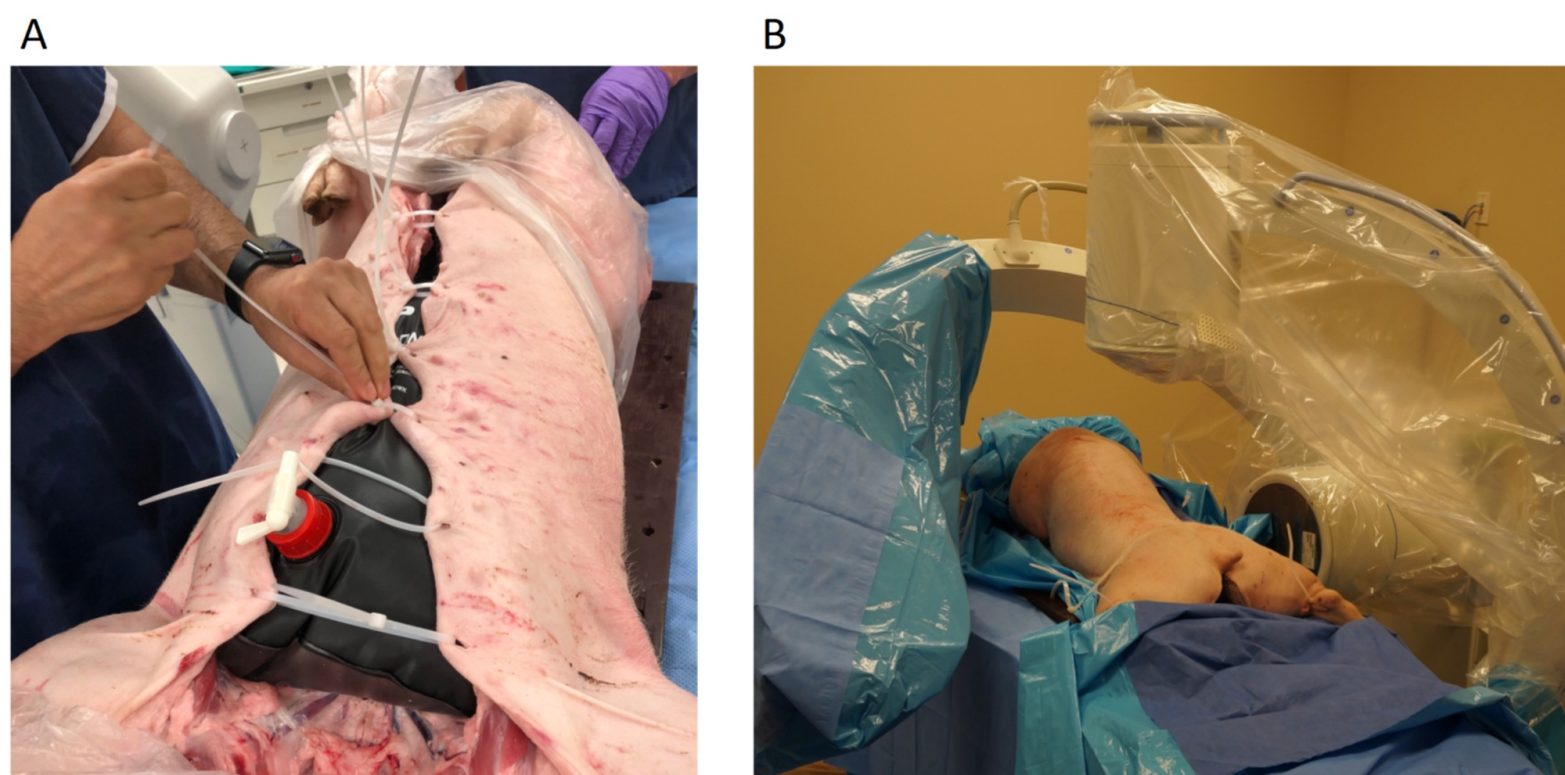
Preparing the cadaver. (A) Filling the abdominal cavity with contrast and water-filled bladder. (B) The porcine cadaver is positioned prone on a custom backboard and secured with cable ties. Anterior-posterior and lateral fluoroscopy are set up for the target level.

Fresh cadavers of male hybrid landrace pigs aged approximately six months weighing 40-70 kg were obtained from a local farmer. The pigs were delivered slaughtered, bled, and gutted which increases the timeframe available for use. To simulate the opacity of the abdominal contents, we placed a rubber bladder (CampShower 5 gallon) filled with 16 liters of water in the abdominal cavity and sealed the abdomen with cable ties (Figure 1A). The water in the bladder can be mixed with contrast (Omnipaque 240 mg/ml, GE Healthcare) to achieve the most realistic level of contrast for the specific application.

**Figure 2:**
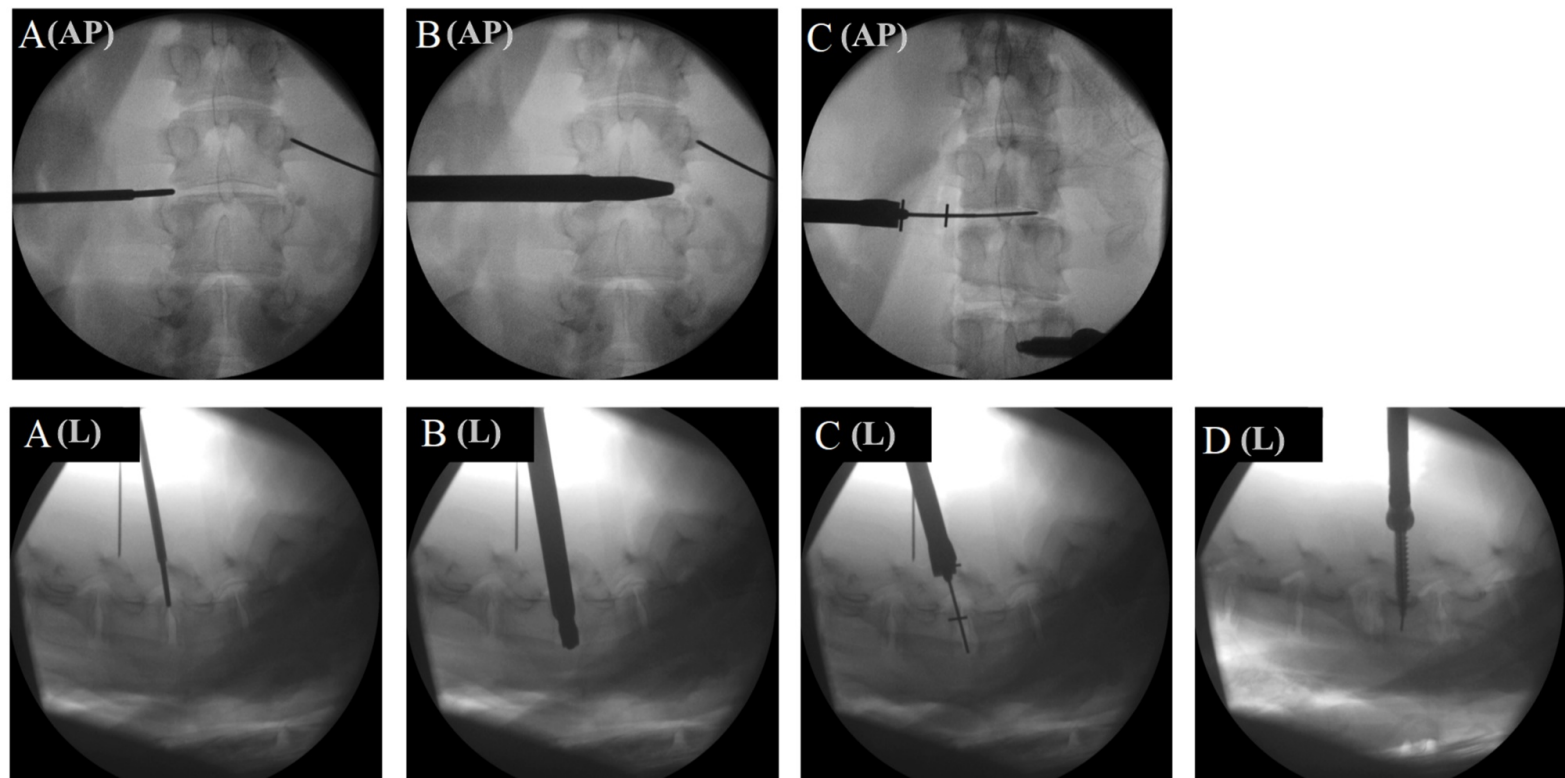
Progress of the procedure under anterior-posterior (AP) and lateral (L) fluoroscopy. (A) Approaching the disc space with blunt electrophysiological probe. (B) Discectomy. (C) Placing the cage into the disk space over a K-wire. (D) Pedicle screw placement.

The instrumentation and OR setup were identical to our routine setup for the OLLIF procedure which has been previously described [2]. We position the cadaver in prone position on a custom wooden backboard with cable ties (Figure 1B). The cadaver is draped in the usual fashion using transparent draping. We set up anterior-posterior (AP) and lateral fluoroscopy for the target level (Figure 1B), so that the endplates of the target level are perpendicular in the lateral view and the spinous process is centered between the pedicles in the AP view. The entire procedure under lateral and AP fluoroscopy is shown in Figure 2.

**Figure 3:**
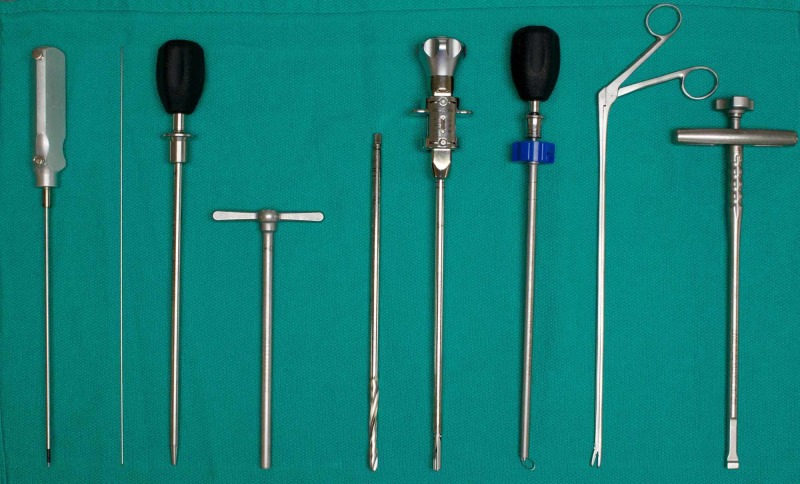
Surgical instruments used in the lab. From left to right: Probe, K-wire, dilator, access portal, drill, rotating curette, rongeur, long pituitary, cage holder

Compared to the approach in humans which is typically performed at 45° to the midline vertical, the porcine spine requires a more lateral approach to compensate for shallower AP diameter of vertebral body. Although electrophysiology cannot be simulated in this model, we still approach the disc with a blunt neurophysiological probe with a sleeve. Once the probe makes contact with the disc, the probe is removed while the sleeve remains in place and a K-wire is inserted. Next we enter a dilator over the K-wire and tap it into the disc space until the tip is past the midline. We then insert an access portal over the probe and perform the discectomy through that portal using a drill, rotating curette, long pituitary, and rongeur (Figure 3). Before removing the access portal, we insert a K-wire to mark the disc space. We enter the cage into the disc space over the K-wire and tap it into the disc space with a mallet. Finally, we perform minimally invasive pedicle screw fixation. Due to the shallower AP diameter of the pedicles, we used smaller pedicle screws, typically 35-40 mm. After the lab, the cadaver is dissected layer by layer to visualize the approach and the effects of the procedure on spinal and neural structures. We subsequently removed soft tissue from the spine by placing the specimen in water at a low boil for 90 minutes, creating bone specimens for studying the anatomy.

Relevant differences were reported between the porcine and human anatomy based on our experience and a review of the literature. We performed this lab five times with eight surgeons and solicited feedback from the participants.

## Results

Anatomical comparison

There are several differences in anatomy that are relevant for practicing minimally invasive fusion on porcine cadavers. The lumbar lordosis in the porcine spine is much lower than in humans. However, we were able to increase the lumbar lordosis by removing the abdominal organs and positioning the cadaver as described above.

Pigs have 14-15 thoracic vertebrae and six lumbar vertebrae. While thoracic vertebrae are substantially different from humans, the lower lumbar vertebrae are most similar to humans [10]. Some key comparisons are represented in Table 1. We found that while the diameter of the vertebral body in the dorsal-ventral dimension is about 70% smaller than in humans, the lateral diameter is very similar.

**Table 1:** Major anatomic differences between porcine and human spinal anatomy. Based on our experience with porcine specimens and Busscher et al. (2010) [10].

Variable for Comparison	Human	Porcine
Number of Thoracic Vertebrae	15 Thoracic	12 Thoracic
Number of Lumbar Vertebrae	6 Lumbar	5 Lumbar
Lumbar Lordosis	29°	Minimal
Thoracic Kyphosis	35°	16°
Dimension of pedicle	Relatively narrower	Relatively wider, but similar height
Vertebral Body Height	Between 20-30 mm	Similar
Intervertebral Disk Height	Approximately 10 mm	Approximately 5 mm
Endplate Dimensions	Width to Depth Ratio 1.5	Width to Depth Ratio 2, appears relatively larger in anterior-posterior dimension

**Figure 4:**
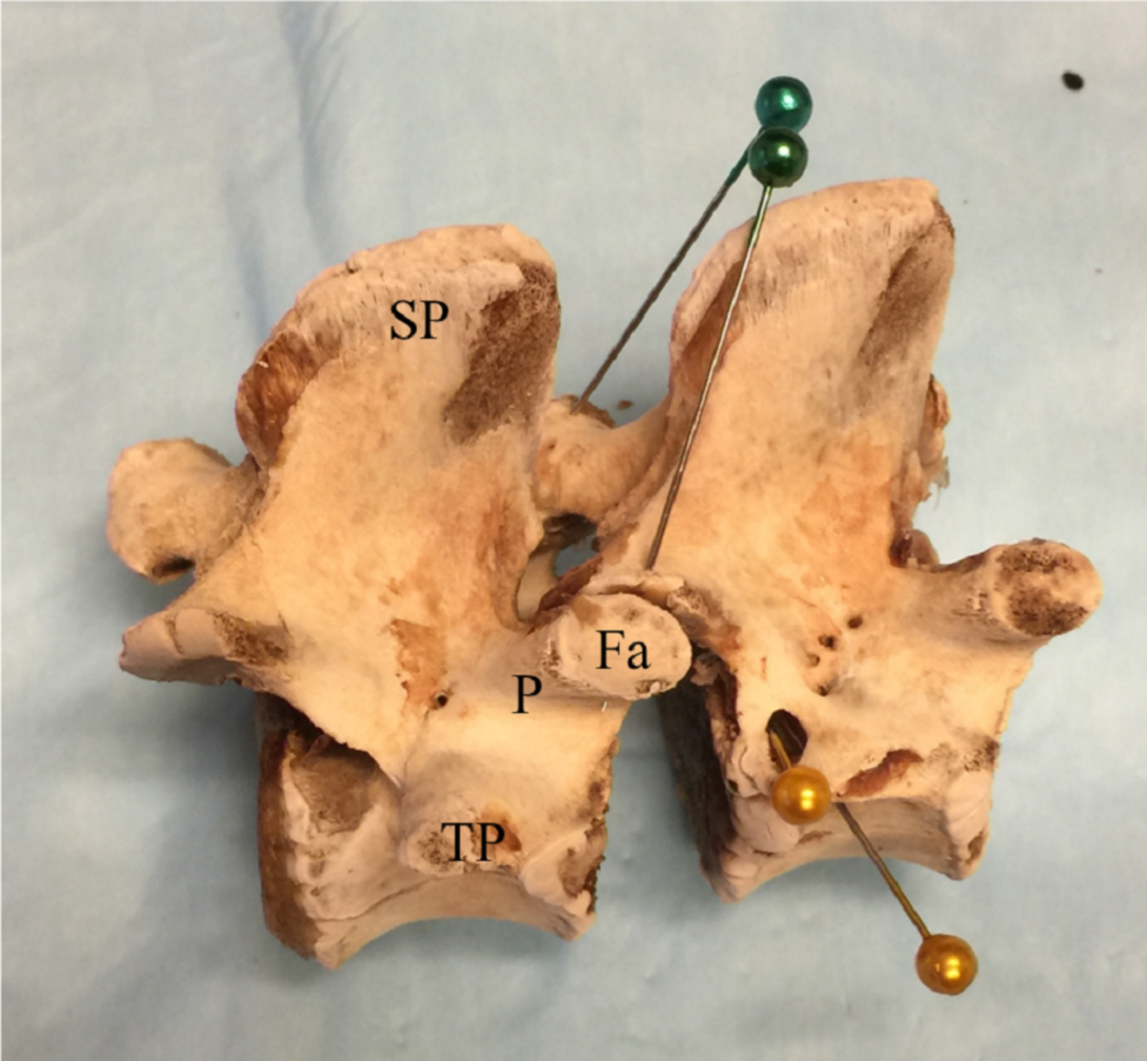
Lateral view of porcine lumbar vertebrae after soft tissue removal. The “neural” foramen where the nerve root exits is marked with the gold pin. SP: Spinous process; P: Pedicle; TP: Transverse process; VB: Vertebral body; Fa: Facet.

In the lower lumbar spine the intervertebral foramen is very similar to humans. In the high lumbar spine the intervertebral foramen becomes bi-lobed and a bony separation appears between the discal foramen and “neural foramen” where the nerve root exits. This neural foramen is marked with a gold pin in Figure 4. The separation of these two foramina becomes more pronounced in the thoracic spine.

**Figure 5:**
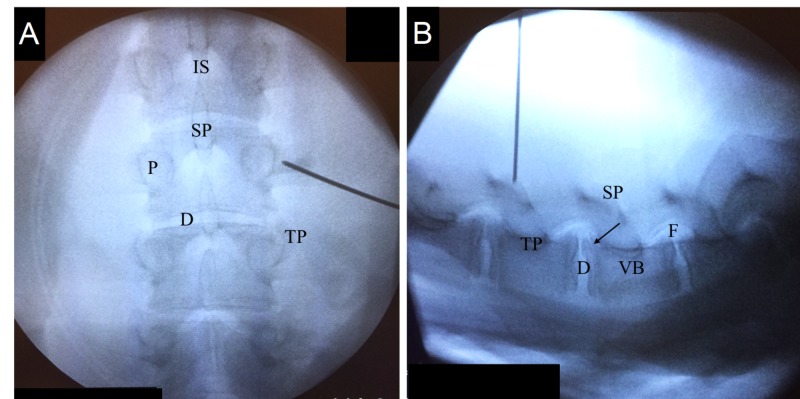
Fluoroscopic view of the porcine spine. (A) Anterior-posterior. (B) Lateral arrow points to osteophytic process of the vertebral body. IS: Interlaminar space; SP: Spinous process; P: Pedicle; D: Disc; TP: Transverse process; VB: Vertebral body; F: Foramen.

We also found that the transverse process is relatively longer than in humans and takes off on the ventral aspect of the pedicle rather than on the dorsal aspect as in humans. In all our specimens, the edge of the vertebral body extends further posteriorly than in humans (arrow in Figure 5). This appears physiologic in pigs but would be labelled an osteophytic change in humans, which are common in patients who undergo spinal surgery for degenerative conditions.

The consistency of the disc in our porcine specimens was similar to the human disc, but the disc appears heart shaped with a concave posterior border compared to human disc which is more oval shaped. Additionally, we noticed that the ossification of the vertebral body in our young specimens had not been completed, which can lead to separation along the epiphysis if the cage is entered with poor trajectory, which can be used as a maker for an adequate trajectory.

We found that the angle, size and configuration of L5/S1 and the SI joint were substantially different in pigs compared to humans, making them unsuitable for training on this level.

Participant feedback

We are well aware of the low number and anecdotal nature of the feedback collected. Nevertheless we gathered such feedback from most of the participants. Our future goal is to expand upon this study to involve a higher number of participants to further evaluate the teaching experience this model provides so as to allow us to assess the utility of this model. Feedback from lab participants was generally positive. Representative quotes from participants are in Table 2. Surgeons noted that the lab allowed them to “get down the approach and the anatomy” and the lab helped them “become more comfortable with the procedure.” Participants noted that the most realistic part of the experience was the “tactile feedback,” and uniformly stated that they preferred the experience to training on synthetic foam models. Participants did note that the anatomy was different compared to humans but also mentioned that because “the specimen was fresh compared to a [preserved] cadaver […] the tactile feedback was more realistic.”

**Table 2:** Narrative feedback provided by lab participants. Feedback obtained from eight surgeons over five labs.

Question	Participant Feedback
What was the most realistic part of the experience compared to humans?	“Tactile Feedback”, “the fact that the anatomy was similar to a human”
What was the least realistic part of the experience compared to humans?	“Anatomy was different but everything else was realistic”, “Only drawback is that the pig anatomy is not exactly identical to human anatomy but as a means of a practice run was very valuable.”
What was the most useful part of the experience?	“Getting down the approach and the anatomy, I thought [the model] worked very well for the approach”
How did the program contribute to your professional development?	“Helped me become more comfortable with the procedure”, “It provided enough encouragement to begin performing the procedure at my practice”
How does the pig lab compare to training labs on foam models?	“Much much better”, “Far better and more realistic than trying out in [synthetic] models”
How does the pig lab compare to training labs on human cadavers?	“Slightly less helpful but only because of the anatomy”, “The pig lab was good in that the specimen was fresh compared to a [preserved] cadaver and the tactile feedback was more realistic.”, “C arm set up was similar as one in cadaver labs”

## Discussion

We found that porcine cadavers have the potential to be an appropriate substitute for human cadaver as a tool for learning minimally invasive spinal procedures of the lumbar spine between L1-L5. Participants in our labs agreed that porcine cadaver models are a valuable learning tool and we now routinely use porcine cadavers in a step-up approach, training surgeons first on porcine cadavers before transitioning to human cadaveric spines and subsequently supervised training in the OR. While we used this model to train surgeons in trans-Kambin lumbar fusion and pedicle screw fixation, the anatomical principles and preparation of the cadaver we described above are generally applicable. While this study was designed only as a proof of concept that using porcine cadavers is feasible, we hypothesize that porcine models can be used to train surgeons in a wide variety of MIS techniques and that doing so will improve surgeons skill and comfort with these techniques. Further study is required to investigate whether porcine models offer a superior training experience compared to other models of MIS training.

Practical experience

Under fluoroscopy, anatomical differences are more apparent in the lateral view compared to the AP view. The transverse processes in pigs appear more pronounced than in humans and they take off on the ventral aspect of the pedicle, so it has the potential to be confused with the facet to inexperienced trainees. The spinous processes point straight dorsally, unlike in humans where they are angled caudally.

Due to the shallower vertebral body, we performed a more lateral approach compared to human spines where we approached the disc at an angle of 45° to the midline. The layers of soft tissue dilated during approach felt very similar compared to humans and the tissue had a similar force-response compared to humans, allowing for dilation along the natural fibers of the tissue. This was noticeably different from synthetic foam models, where the anatomical layers are absent and dilation does not reflect the tactile feedback obtained from biological tissues. However, the para-spinal muscles are significantly larger than in humans which means the approach distance corresponds approximately to a far lateral approach in humans. Upon entry into the disc space, we clearly felt the penetration of the disc capsule. During the subsequent discectomy, the capsule acts as a fulcrum to stabilize the instruments, as is the case in humans. Again, this effect is entirely absent in foam models in which the disc has a uniform consistency.

In our specimens, the cartilaginous area of the disc appeared more pronounced than in humans, most likely due to the age of the pig. This allowed for a more extensive discectomy and end plate preparation than is possible in humans, which is beneficial for training purposes. Cage entry required less force compared to humans which may be related to increased anatomical flexibility of the young pigs used in this lab. We used cages sized 27 mm in length to accommodate the shallower vertebral body, while cage height was between 9-11 mm. We found that the porcine pedicle is an excellent model for practicing pedicle screw placement, because the pedicle is larger in the cranial-caudal and lateral dimension, allowing for easy pedicle identification. The size and trajectory of the pedicles was sufficiently similar to humans to use the same landmarks for navigation during pedicle screw placement. However, the pedicles are relatively shallower in the AP dimension, requiring the use of shorter pedicle screws between 35-40 mm.

Advantages of porcine cadavers

Biological specimens like porcine cadavers have several advantages over foam models. Biological specimens have naturally variable anatomy which gives surgeons useful experience in navigation and adapting to each patient’s unique anatomy. This is a significant advantage over synthetic foam models, which have uniform anatomy and appear almost cartoonish on fluoroscopy. After performing the procedure, surgeons are able to dissect the porcine cadaver to better understand the anatomy, approach trajectory, and effect of the procedure on the tissues. Porcine cadavers are especially useful for training surgeons on procedures that rely on tactile feedback during approach. The force-response of different soft tissue layers closely resembled our experience in human patients. Once the disc is reached, the presence of a disc capsule makes discectomy much more realistic than is possible in synthetic models. Due to the distinct layers of the porcine disc, surgeons were able to gain experience in using the specialized tools required for trans-Kambin discectomy. We also found that the porcine spine is a valuable tool for introducing pedicle screw fixation techniques due to the more prominent pedicles.

The main advantage of the porcine models is their low cost and widespread availability. Porcine cadavers are widely available and cost around $180 compared to $500 for foam models and, $5,000-10,000 for human cadavers. This makes porcine cadavers a particularly appealing option for training surgeons in developing countries where cost and availability are an even more important consideration. If proper handling is observed and sterile tools are observed, the porcine cadaver can be donated for further use following the completion of the training course. We recommend using porcine cadavers of weighing 40 to 60 kg, between six to eight months of age. Older pigs often have ossified spinal ligaments and bulkier vertebral bodies [11], while smaller cadavers are too small to be a realistic model for training. We recommend cadavers that have been gutted and bled both because it makes the lab more sanitary and because some lumbar lordosis can be generated by fixing a gutted porcine cadaver on a flat surface.

Disadvantages of porcine cadavers

The main drawbacks of using porcine cadavers are differences in the anatomy, as was noted by our anatomical study. These differences are related to the fact that the porcine spine bears lower loads in the cranial-caudal direction compared to the human spine. For the purpose of surgical training, the most meaningful anatomical difference that we encountered was shallower VB diameter, which requires adjustment of the cage trajectory for a more lateral approach. Additionally, the porcine spine is a poor model for L5-S1 because of the angle, configuration and size of the sacral ala and iliac crest. Porcine cadavers are also a poor model for sacro-illiac fusions because the anatomy is substantially different from humans.

## Conclusions

Fresh pig cadavers are a useful tool for training surgeons to perform minimally invasive spine surgery. Although certain anatomical differences must be taken into account, porcine cadavers are an especially useful tool for training surgeons in procedures where tactile feedback is key to the success of the operation. Fresh porcine cadavers are a far more realistic training model than synthetic models, are less expensive, and can be handled more easily than human cadavers. Using porcine cadavers for the initial stages of training may increase the availability of training courses in MIS surgery. Further study is required to investigate whether porcine cadavers offer a superior training experience compared to other MIS training models.
